# Supplementary material for: Blue light and CO2 signals converge to regulate light-induced stomatal opening
Source: Nat Commun. 2017 Nov 3;8:1284. doi: 10.1038/s41467-017-01237-5 (PMC5670223; doi:10.1038/s41467-017-01237-5)
Supplement: Supplementary file 1 — Supplementary Information [file 41467_2017_1237_MOESM1_ESM.pdf]

Supplementary Fig.1

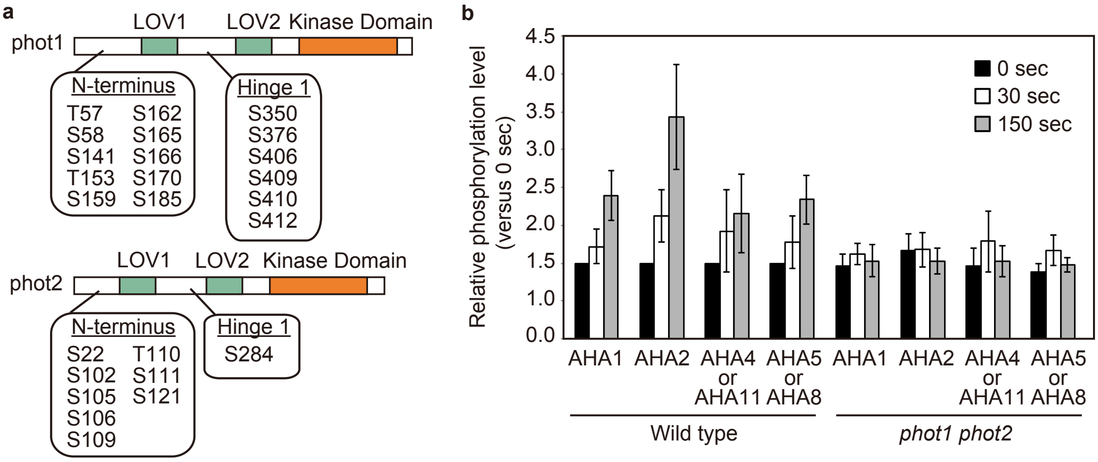

Supplementary Figure 1. Phosphorylation sites in phototropins and the H^+-ATPases. (**a**) Autophosphorylated amino acid residues in phot1 and phot2 in response to BL in guard cells. Ten sites in the N-terminal region and five sites in the hinge region were phosphorylated. Among these 15 sites, 13 sites were phosphorylated in response to blue light. Four new phosphorylation sites (S141, T153, S406, and S409) were found. We also found that eight sites in phot2, including seven sites in the N-terminal region and one in the hinge region were phosphorylated. Only S102 was newly found. (**b**) Phosphorylation of H^+-ATPases in guard cells that express 11 isoforms in response to BL. Among these isoforms, the penultimate Thrs of AHA1, 2, 4 (or 11), and 5 (or 8) were phosphorylated in a phototropin-dependent manner, and the phosphorylation levels increased with the time after BL treatment. Continuous RL (600 μmol m^{-2} s^{-1}) was applied to GCPs for 30 min and the BL pulse (100 μmol m^{-2} s^{-1}, 30 s) was superimposed.

Supplementary Fig.2

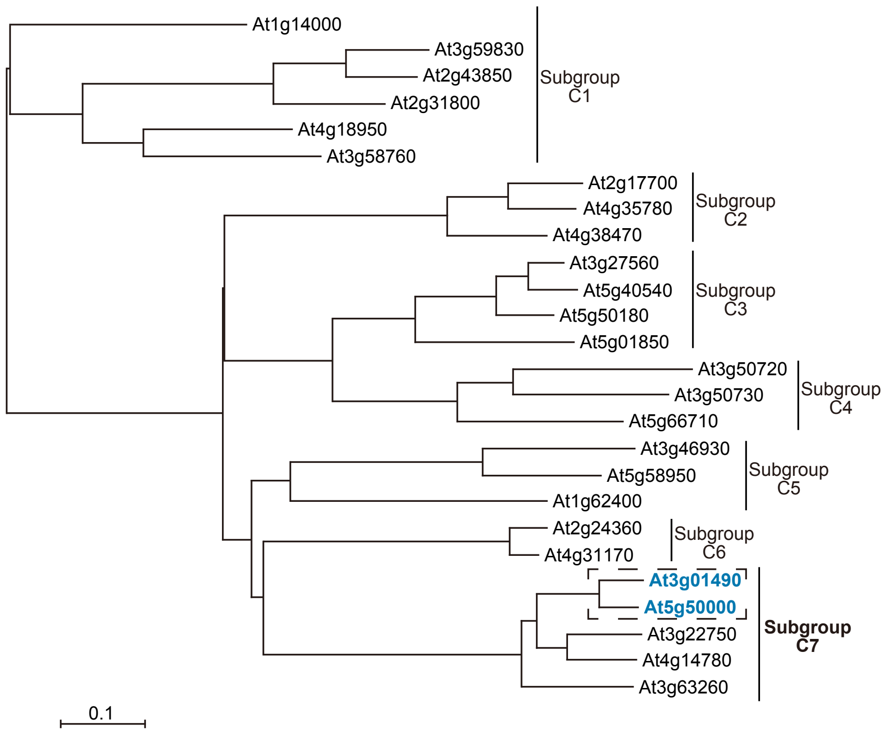

Supplementary Figure 2. Phylogenetic tree of group C MAPKKKs. Phylogenetic analysis was done using the MEGA software version 6. Genes of group C MAPKKKs were referred to ref.53. The scale bar corresponds to 0.1 substitutions per site.

.

Supplementary Fig.3

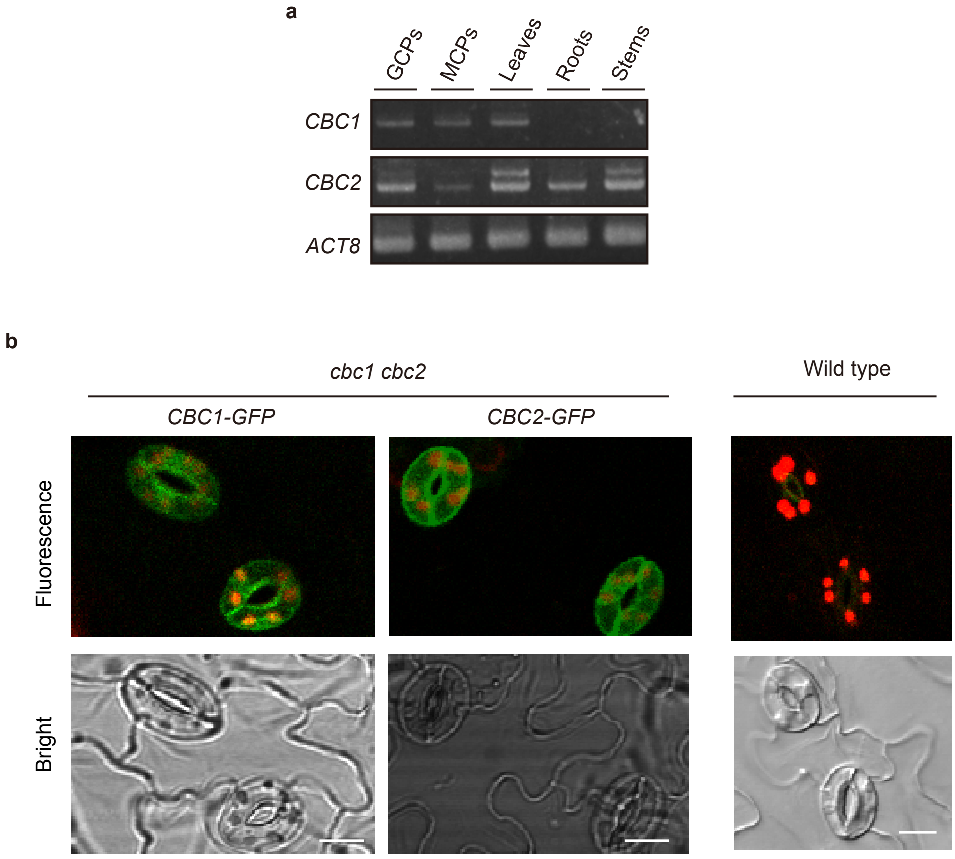

Supplementary Figure 3. **Expression of *CBC1* and *CBC2* genes**. (**a**) RNAs were extracted from GCPs, MCPs, leaves, roots, and stems using ISOGEN (Nippon Gene) and RNeasy (Qiagen). cDNAs were synthesized from the RNA extracts and, PCR experiments were done using the specific primers for *CBC1* and *CBC2*. (**b**) Expression of *CBC1-GFP* and *CBC2-GFP* using each own promoter in guard cells in the epidermal peels. GFP-fluorescence was determined using a confocal laser-scanning microscope (Digital Eclipse C1; Nikon). White bars represent 10 μm.

Supplementary Fig.4

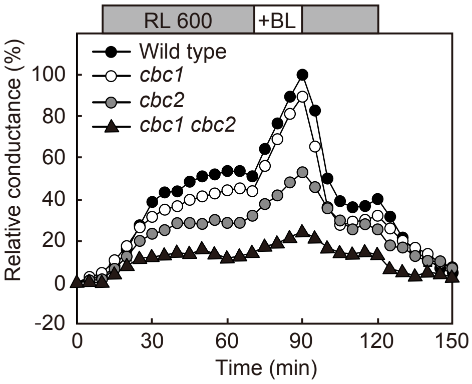

Supplementary Figure 4. Light-induced stomatal opening determined by stomatal conductance in leaves. Stomatal conductance increases in WT, *cbc1*, *cbc2*, and *cbc1 cbc2* in response to RL and BL superimposed RL were normalized to the same starting values on the basis of the data presented in Fig. 1**i,** and were expressed as relative values.

Supplementary Fig.5

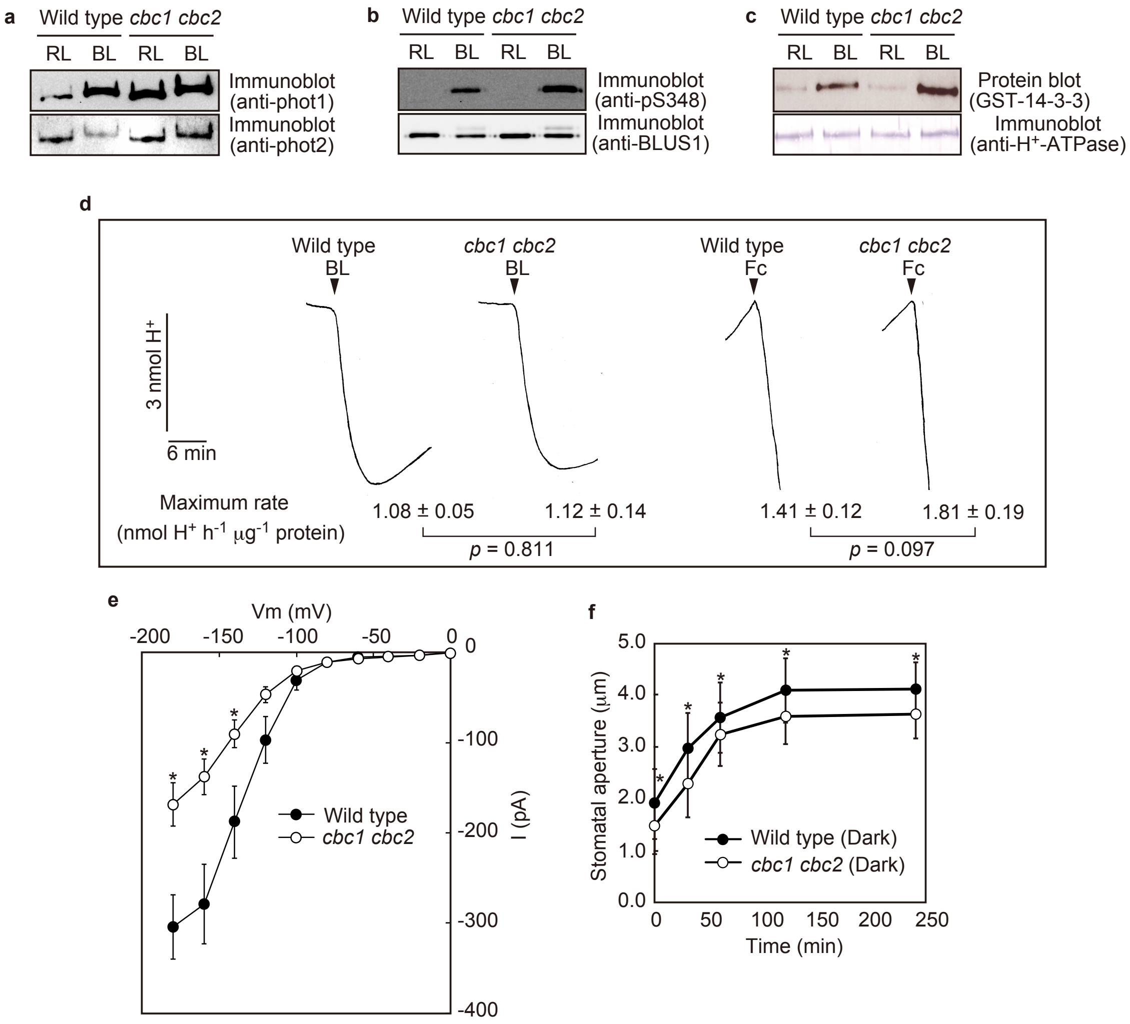

Supplementary Figure 5. **Phototropin-mediated activation of H^+-ATPases was not impaired in the *cbc1 cbc2* double mutant**. (**a**) Autophosphorylation of phot1 (upper panel) and phot2 (lower panel) in GCPs from *cbc1 cbc2*. (**b**) BL-dependent BLUS1 phosphorylation in GCPs was visualized using anti-pS348 antibodies. The protein amount (lower panel) was determined using anti-BLUS1 antibodies. (**c**) BL-dependent phosphorylation of a H^+-ATPase in GCPs from *cbc1 cbc2*. H^+-ATPase phosphorylation was indirectly measured by 14-3-3 protein binding. (**d**) BL- and Fc-dependent H^+-pumping from GCPs. Means with ± s.e.m. (**e**) Voltage-dependent whole-cell K^+_{in}

current in GCPs from WT (*n* = 6 experiments) and *cbc1 cbc2* (*n* = 7 experiments). Bars represent ± s.e.m. (* $P < 0.05$). The voltage protocol was stepped up from 0 to −180 mV in 20 mV decrements (holding potential, −40 mV). (**f**) Fc-induced stomatal opening in epidermal peels of WT and *cbc1 cbc2*. The peels were treated with 10 μM Fc and incubated in the dark. Stomatal apertures were measured in the basal reaction mixture at the indicated times. Data are means ± s.d. (*n* = 75).

Supplementary Fig.6

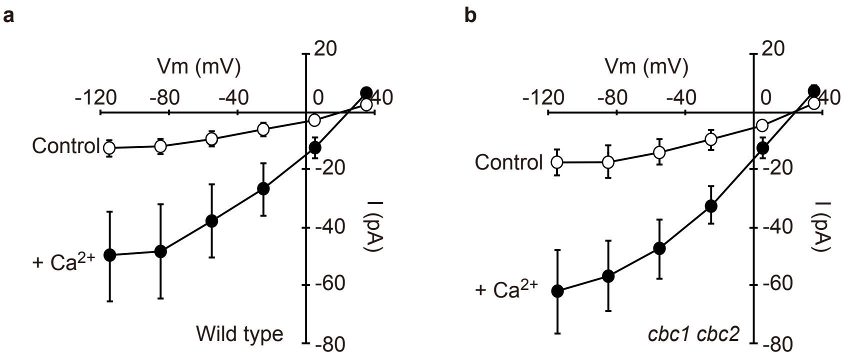

Supplementary Figure 6. The S-type anion channel activity in GCPs from WT plants and the *cbc1 cbc2* double mutant. We performed whole cell patch-clamp analysis of GCPs from WT plants (a) and the *cbc1 cbc2* double mutant (b) in the presence of 2 μM $[Ca^{2+}]_{cyt}$ with 40 mM extracellular Ca^{2+} under dim white light. No difference of the S-type anion current was found in GCPs between WT plants and the *cbc1 cbc2* mutant in the absence and presence of Ca^{2+}.

Supplementary Fig.7

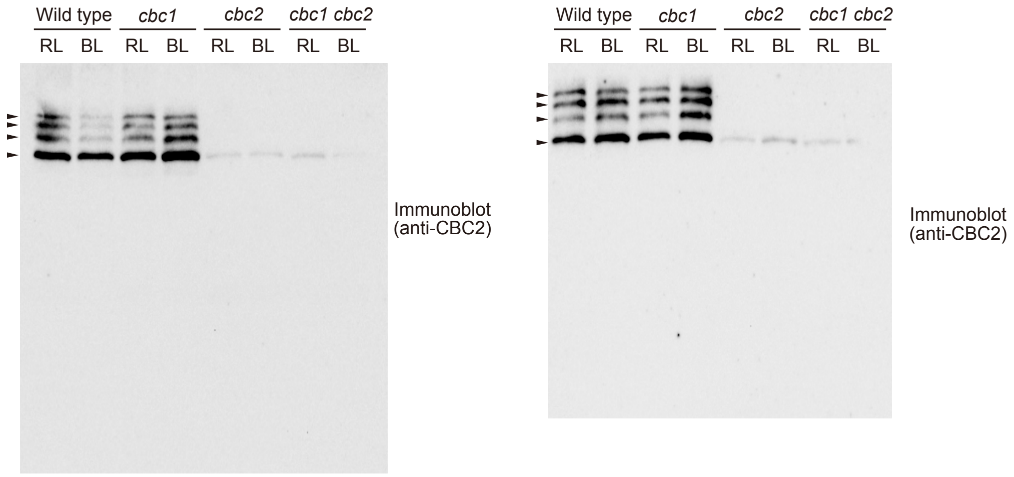

Supplementary Figure 7. Immunoblot analysis of CBC2. The analysis was performed after electrophoresis on Phos-tag SDS-PAGE as Fig.4a. GCPs were illuminated with light as described in Fig.1**a.** Samples were obtained 1 min after the start of the BL pulse.

Supplementary Fig.8

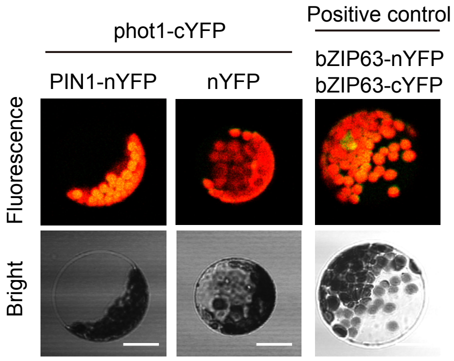

Supplementary Figure 8. BiFC assay of phot1 and PIN1. PIN1-nYFP and phot1-cYFP were co-transfected to MCPs. bZIP63-nYFP and bZIP63-cYFP were co-transfected to MCPs as a positive control. White bars represent 20 μm.

Supplementary Fig.9

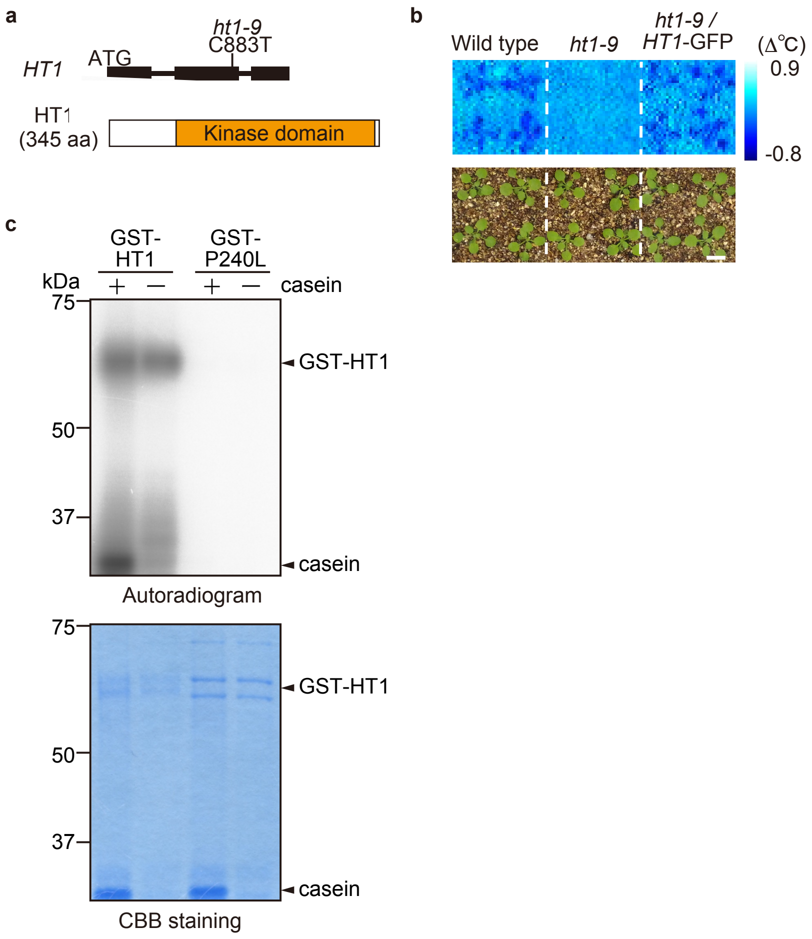

Supplementary Figure 9. Identification of *ht1-9* as a kinase-dead mutant. (**a**) Genomic and protein structures of *HT1* and the site of the *ht1-9* mutation. Mutation of C883T results in the conversion of Pro 240 to Leu in the kinase domain of HT1.

(**b**) Complementation of the BL-dependent temperature decrease by transforming *ht1-9* with *HT1* cDNA. Upper: the subtracted image for leaf temperature. Lower: photo of plants. (**c**) Upper: Autophosphorylation and substrate phosphorylation of HT1 and *ht1-9* (P240L). Lower: CBB staining of GST-HT1 and casein substrate. The GST proteins were expressed in *E. coli* and purified with glutathione-Sepharose beads.

Supplementary Fig.10

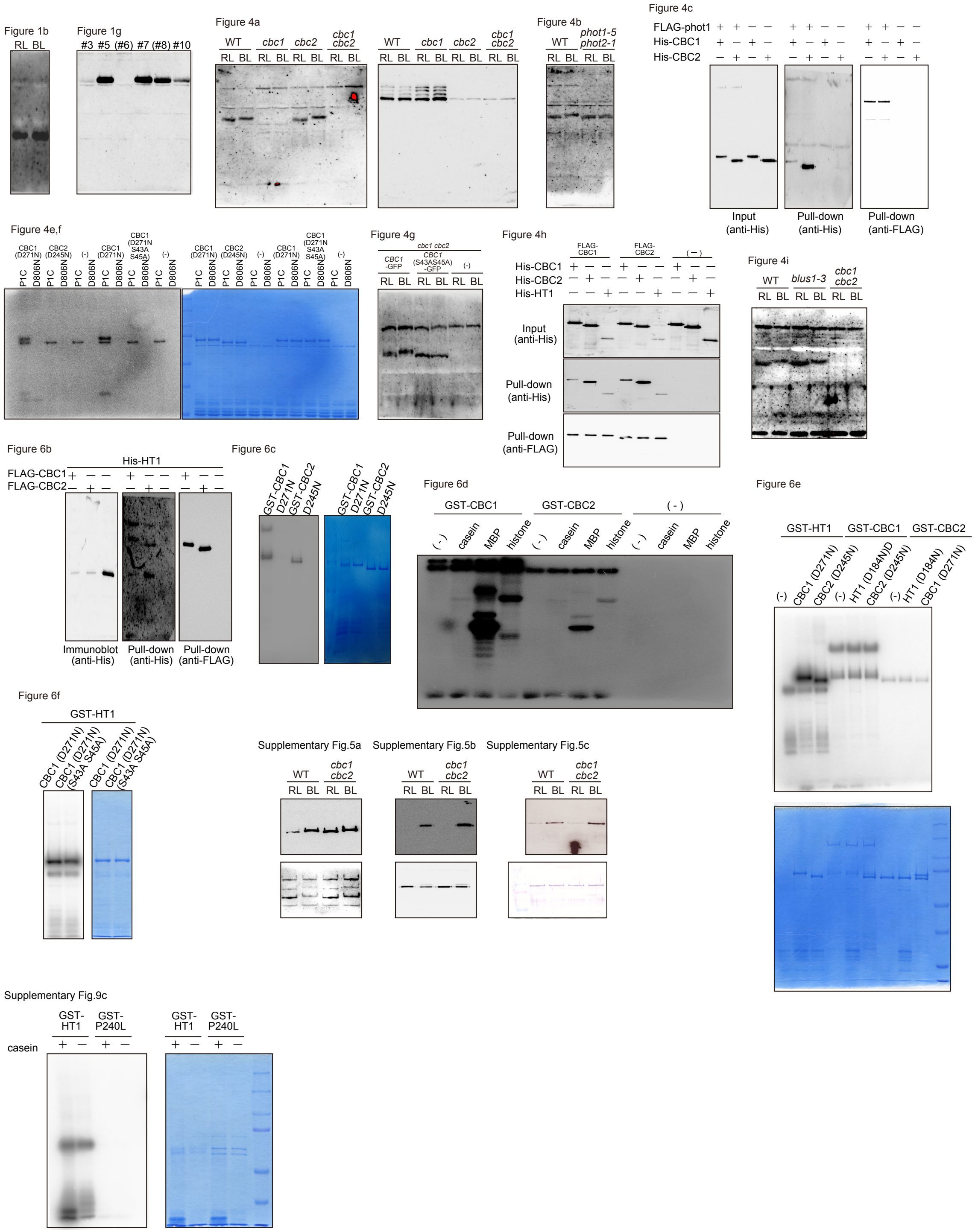

Supplementary Figure 10. Uncropped pictures of western blots presented in this study.

Supplementary Table 1

a

Plant materials	Stomatal aperture (μm)	
Wild type	2.89 ± 0.19	*
cbc1 cbc2	2.55 ± 0.17	

b

Plant materials	Stomatal width (μm)	Stomatal length (μm)
Wild type	14.94 ± 1.20	20.94 ± 1.57
cbc1 cbc2	14.75 ± 1.03	21.42 ± 1.59

Supplementary Table 1. Stomatal apertures in the dark (a), and sizes of stomata (b), in WT plants and the *cbc1 cbc2* double mutant. (a) Data represent means ± s.e.m., *n* = 300, from Fig.1**h**, Fig. 2**c**-**e**) (**b**) Data represent means ± s.d., *n* = 75, triplicate experiments. (**a, b**) Asterisks denote the significant differences in stomatal apertures between WT plants and mutants under darkness. *P < 0.05 by Student's *t* test.

Supplementary Table 2

Plant materials	Δ Stomatal conductance to H_2O (mol H_2O m^{-2} s^{-1})		Relative conductance change (versus Wild type)	
	RL	BL	RL	BL
Wild type	0.053 ± 0.013	0.051 ± 0.009	1.00	1.00
cbc1	0.046 ± 0.011	0.048 ± 0.020	0.86	0.94
cbc2	0.033 ± 0.015*	0.022 ± 0.013*	0.63	0.43
cbc1 cbc2	0.015 ± 0.009*	0.011 ± 0.009*	0.28	0.21

Supplementary Table 2. RL-induced and BL-dependent stomatal conductance changes. Quantification was done on samples prepared as described in Fig. 1**i**. The magnitude of RL-induced stomatal conductance increase was obtained from the steady state levels of conductance. The magnitude of BL-dependent conductance increase was obtained by subtracting the RL-induced conductance from the peak values after BL irradiation. Experiments were done eight times, and the average values were presented with the standard errors. Relative values were also shown in the two right columns. Asterisks denote the significant differences in conductance changes in intact leaves between WT plants and mutants in response to RL and BL superimposed on the RL. *$P < 0.05$ by Student's *t* test.

Supplementary Table 3

Time after the CO_2 concentration decrease (min)	Δ Stomatal conductance to H_2O (mol H_2O m^{-2} s^{-1})		Relative conductance change (versus RL+ BL)	
	RL + BL	RL	RL + BL	RL
+ 10 min	0.034 ± 0.006	0.024 ± 0.006	1.00	0.71
+ 30 min	0.094 ± 0.011	0.070 ± 0.003*	1.00	0.75
+ 50 min	0.128 ± 0.011	0.100 ± 0.004*	1.00	0.78

Supplementary Table 3. Stomatal conductance increases in response to the CO_2 concentration decrease under RL (90 μmol m^{-2} s^{-1}) plus BL (10 μmol m^{-2} s^{-1}) or RL (100 μmol m^{-2} s^{-1}). Stomatal conductance increases were obtained by subtracting a conductance determined before the CO_2 concentration decrease from ones determined at 10, 30, and 50 min depicted in Fig. 5**d.** Larger conductance increase in response to the low CO_2 was found under RL plus BL than under RL. Asterisks denote the significant differences in conductance changes in intact leaves between WT plants under RL plus BL or RL. *P < 0.05 by Student's *t* test.

Supplementary Table 4

	Gene name	site	Fw primer(5'−3')	Rv primer(5'−3')
cloning primer	*CBC1*	promoter+5'−UTR+gene	CAGAACCAACCCTGCTTTGTACG	TGGGCCTCGGTGTCGGCG
		3'−UTR	AGTACACACACATGCACTACGTTCC	CTGCGGAAGGATTTATCGATC
		cds	ATGAAGGAGAAGGCGGAGAGTG	TCATGGGCCTCGGTGTCG
	CBC2	promoter+5'−UTR+gene	GTCAGTAAGAAAAGTAGTGAAAGTATCC	AGGACCACGTTTCCTTCGGAAG
		3'−UTR	TTCCCAAAGACCAATCTAAGATC	TGATGAGGAAGGAGCAGAGGGG
		cds	ATGAAAGAAGGAAAGGATGGGTTTG	TTAAGGACCACGTTTCCTTCG
RT−PCR	*CBC1*		GAGAGTGGTGGAGGAGTAGGATAC	TCATGGGCCTCGGTGTCG
	CBC2		ATGAAAGAAGGAAAGGATGGGTTTG	TTAAGGACCACGTTTCCTTCG
	ACT8		ACTTTACGCCAGTGGTCGTACAAC	AAGGACTTCTGGGCACCTGAATCT
Site−directed mutagenesis	*CBC1 (S43A S45A)*		GGACGATGGAGAAGAGGAAGgcTTTGgcTGATGGTGAAGATAAC	GTTATCTTCACCATCAgcCAAAgcCTTCCTCTTCTCCATCGTCC

Supplementary Table 4. Primers used in this study.
