# Supplementary material for: Blue light and CO2 signals converge to regulate light-induced stomatal opening
Source: Nat Commun. 2017 Nov 3;8:1284. doi: 10.1038/s41467-017-01237-5 (PMC5670223; doi:10.1038/s41467-017-01237-5)
Supplement: Supplementary file 3 — Description of Additional Supplementary Files [file 41467_2017_1237_MOESM3_ESM.pdf]

**Description of Additional Supplementary Files**

File Name: Supplementary Data 1
Description: Phosphopeptides in guard cells in response to blue light.
